# Recent Developments in Ion Channel and Ion-Related Signaling

**DOI:** 10.3390/ijms241914419

**Published:** 2023-09-22

**Authors:** Susumu Ohya

**Affiliations:** Department of Pharmacology, Graduate School of Medical Sciences, Nagoya City University, Nagoya 467-8601, Japan; sohya@med.nagoya-cu.ac.jp; Tel.: +81-52-853-8149

Ion channels play an important role in the cellular functions of various organ systems, such as the nervous, cardiovascular, immune, and endocrine systems, and are potential therapeutic targets for treatments of their dysfunctions, via ‘channelopathy’ [1]. Ion channels modulate diverse intracellular signaling pathways involved in neuronal activity, muscle contraction, cell proliferation, differentiation, apoptosis, and transcription. In addition, ion channel regulatory proteins alter the electrophysiological characteristics, cellular localization, membrane trafficking, and drug sensitivity of ion channels, and contribute to functional diversity and cell-specific responses. Organellar ion channels in the endoplasmic/sarcoplasmic reticulum and mitochondrial inner membrane are also the focus of new therapeutic targets in cellular function and dysfunction [2].

This Special Issue of the *International Journal of Molecular Sciences* (Biochemistry section), entitled “Recent Developments in Ion Channel and Ion-Related Signaling”, included a total of nine contributions, comprising seven original articles and two reviews, which provided new information on the recent advances in research on ion channels and their regulatory molecules.

Yuan et al. [3] investigated the molecular mechanisms underlying heparin-induced sperm capacitation and fertilization. Olfactory receptors (ORs) belonging to the G-protein-coupled receptor family are primarily located in olfaction tissues; however, they are also located in non-olfactory tissues, including sperm, and are involved in sperm capacitation. The activation of OR induces the influx of cellular Ca^2+^, leading to the modulation of motility and chemotaxis in sperm, and the Ca^2+^ influx is a prerequisite for sperm capacitation. They indicated the interactions between heparin and OR2C1, and determined the heparin-binding sites of OR2C1 through molecular dynamics simulation: Arg3, Ala6, Thr7, Asn171, Arg172, Arg173, and Pro287. OR2C1 was up-regulated in the heparin stimulation, and a knockdown of OR2C1 inhibited sperm capacitation. They concluded that OR2C1 plays a potential role in heparin-induced sperm capacitation.

He et al. [4] investigated the involvement of the chordate NFAT5 in the regulation of lumen osmotic pressure via an ion channel-mediated pathway on luminal organ formation. Changes in the apical delivery of ions by ion pumps and channels create an osmotic pressure within the lumen of acini. They indicated that (1) the expression level of *Ciona* NFAT5 (Ci-NFAT5) increased during notochord lumen formation and expansion, (2) a knockdown of NFAT5 in *Ciona* embryos impaired the notochord lumen expansion, and (3) the Ci-NFAT5 transferred from the cytoplasm into nuclei under the hyperosmotic stimuli. Importantly, Ci-NFAT5 regulated a SLC26 family anion transporter, SLC26A6. Taken together, Ci-NFAT5 responding to hypertonic stress regulates notochord lumen formation and expansion via SLC26A6.

The chemical compound JT010 (2-chloro-N-(4-(4-methoxyphenyl)thiazol-2yl)-N-(3-methoxypropyl-acetamide) is known as a potent and selective activator of TRPA1 (transient receptor potential, ankyrin repeat), a Ca^2+^-permeable cation channel. Matsubara et al. [5] showed that the JT010 activated the ‘human’ TRPA1 (hTRPA1) but not the ‘murine’ one (mTRPA1). They determined that two amino acid residues, cysteine at 621 (C621) and phenylalanine at 669 (F669), are critical for JY010-induced hTRPA1 activation.

Ohya et al. [6] investigated whether the Ca^2+^-activated K^+^ channel (K_Ca_3.1) in tumor-associated macrophages (TAMs) regulated the expression of pro-tumorigenic cytokines and angiogenic growth factors. In THP-1-derived M_2_ macrophages, the expression levels of interleukin (IL)-8 and IL-10 were significantly decreased by pharmacological activation of K_Ca_3.1, without changes in those of vascular endothelial growth factor (VEGF) and transforming growth factor (TGF)-β1. Furthermore, under in vitro experimental conditions that mimic extracellular K^+^ levels in the tumor microenvironment, IL-8 and IL-10 levels were both significantly elevated, and their increases were reversed by K_Ca_3.1 activation. Among several signaling pathways potentially involved in the transcriptional regulation of IL-8 and IL-10, respective treatments with extracellular signal-regulated kinase (ERK) and c-Jun N-terminal kinase (JNK) inhibitors significantly repressed their transcriptions, and activation of K_Ca_3.1 significantly reduced the phosphorylated ERK, JNK, c-Jun, and cAMP response element binding protein (CREB) levels. Taken together, the K_Ca_3.1 activator may suppress IL-10-induced tumor immune surveillance escape and IL-8-induced tumorigenicity and metastasis by inhibiting their production from TAMs through ERK-CREB and JNK-c-Jun cascades.

Solorza et al. [7] reported that spontaneous glycinergic currents in the central amygdala (CeA) are modulated by IL-1β. IL-1β has a dual effect, in that it increases glycinergic current amplitude by affecting glycine receptor (GlyR) channel opening and it reduces the amplitudes by disaggregating glycinergic clusters in normal conditions. Oliva et al. [8] investigated the glycinergic transmission in CeA slices and its modulation by IL-1β under ‘neuropathic pain’. The glycinergic spontaneous inhibitory currents (sIPSCs) of the neuropathic pain model showed a bimodal (small and large) amplitude distribution, different from the normal distribution. The exposure to IL-1β reduced both sIPSC populations but with a more substantial effect on the population with larger current amplitudes. They suggest a possible role for CeA GlyRs in pain processing and in the neuroinflammatory modulation of pain perception.

A Ca^2+^-permeable cation channel, namely transient receptor potential vanilloid 4 (TRPV4), plays a key role in endocytosis. Li et al. [9] investigated the contribution of TRPV4 to exocytosis in melanoma, showing that the TRPV4 agonist promoted prominent vesicle priming from the endoplasmic reticulum, leading to cell ferroptosis. They also identified interactions between TRPV4 and folding/vesicle trafficking proteins that were triggered by Ca^2+^ entry through activated TRPV4. The enhanced TRPV4-mediated activation of folding and vesicle trafficking proteins promoted exocytosis. Taken together, this study uncovered the role of TRPV4 in mediating exocytosis and ferroptosis.

The large-conductance Ca^2+^-activated K^+^ (BK/BK_Ca_/MaxiK/K_Ca_1.1) channel is activated by nitric oxide (NO) and NO-donors in vascular smooth muscle cells, by direct action of NO and cGMP-dependent protein kinase (PKG)-mediated action. Gagov et al. [10] investigated the degree of the contribution of PKG to the NO-induced activation of BK channels in freshly isolated smooth muscle cells from rat tail arteries using the patch clamp technique. They concluded that NO released from NO-donors stimulates the BK currents only through the activation of PKG in rat tail arterial myocytes.

The review by Tureckova et al. [11] summarized the current knowledge of multifunctional and broadly expressed TRPV4 channels. TRPV4 channels play a role in various physiological and pathological cellular processes, such as proliferation, osmoregulation, and oxidative stress, and also in a range of neurological diseases, such as neurodegenerative diseases, strokes, epileptic seizures, pain, and trauma. Astrocytic TRPV4 channels are responsible for the modulation of neuronal excitability, the control of blood flow, and brain edema formation, resulting in cerebral ischemia. They addressed updated and integrated information on TRPV4 channels and their expression in healthy and injured neural cells, with a particular focus on their role in ischemic brain injury.

Dysregulated TRP channels (namely TRPC (Canonical), TRPV (Vanilloid), TRPM (Melastatin), TRPP (Polycystin), TRPML (Mucolipin), TRPN (NO-mechano-potential), and TRPA (Ankyrin)) are linked to many hereditary human diseases [12]. The review by Guo et al. [13] summarized the expressions and functions of TRP channels (mainly TRPCs, TRPVs, TRPMs, and TRPA1) in stem cells, including cancer stem cells. They especially focused on the potential roles of TRP channels regulating stem cell physiology and pathophysiology.
